# Efficiency of an intervention package for arterial hypertension comprising telemanagement in a Cameroonian rural setting: The TELEMED-CAM study

**DOI:** 10.11604/pamj.2013.15.153.2655

**Published:** 2013-08-29

**Authors:** Samuel Kingue, Prisca Angandji, Alain Patrick Menanga, Gloria Ashuntantang, Eugene Sobngwi, Rosemonde Akindes Dossou-Yovo, Francois Folefack Kaze, André Pascal Kengne, Anastase Dzudie, Pierre Ndobo, Walinjom Muna

**Affiliations:** 1 Service of Medicine A, General Hospital of Yaounde, Yaounde, Cameroon; 2 Diabetes and Endocrine service, Yaounde Central Hospital, Yaounde, Cameroon; 3 CHU de Cotonou, Benin; 4 NCRP for Cardiovascular and Metabolic Disease, South African Medical Research Council & University of Cape Town, Cape Town, South Africa, Department of Medicine, Faculty of Health Sciences, University of Cape Town, Cape Town, South Africa; 5 Service of Internal Medicine, General Hospital of Douala, Douala, & Buea faculty of Health Sciences, Buea, Cameroon, Department of Medicine, Faculty of Health Sciences, University of Cape Town, Cape Town, South Africa

**Keywords:** Hypertension, control, telemedicine, Cameroon, sub-saharan Africa

## Abstract

**Introduction:**

Sub-Saharan Africa has a disproportionate burden of disease and an extreme shortage of health workforce. Therefore, adequate care for emerging chronic diseases can be very challenging. We implemented and evaluated the effectiveness of an intervention package comprising telecare as a mean for improving the outcomes of care for hypertension in Rural Sub-Saharan Africa.

**Methods:**

The study involved a telemedicine center based at the Yaounde General Hospital (5 cardiologists) in the Capital city of Cameroon, and 30 remote rural health centers within the vicinity of Yaoundé (20 centers (103 patients) in the usual care group, and 10 centers (165 patients) in the intervention groups). The total duration of the intervention was 24 weeks.

**Results:**

Participants in the intervention group had higher baseline systolic (SBP) and diastolic (DBP) blood pressure, and included fewer individuals with diabetes than those in the usual care group (all p < 0.01). Otherwise, the baseline profile was mostly similar between the two groups. During follow-up, more participants in the intervention groups achieved optimal BP control, driven primarily by greater improvement of BP control among High risk participants (hypertension stage III) in the intervention group.

**Conclusion:**

An intervention package comprising tele-support to general practitioners and nurses is effective in improving the management and outcome of care for hypertension in rural underserved populations. This can potentially help in addressing the shortage of trained health workforce for chronic disease management in some settings. However context-specific approaches and cost-effectiveness data are needed to improve the application of telemedicine for chronic disease management in resource-limited settings.

## Introduction

Despite the heavy burden of communicable diseases in Sub Saharan Africa, cardiovascular diseases are becoming a growing concern. According to the World Health Report 2002, cardiovascular disease accounted for 9.2% of total deaths in the African region in 2001, with hypertension, stroke, cardiomyopathies and rheumatic heart disease being the most prevalent causes [1]. Cardiovascular disease has higher mortality in developing countries than in developed ones [2], and disproportionately affects younger people and women. Hypertension remains the most threatening CVD risk factor in the region, with prevalence rates ranging 15% to 30% among adults [3]. Extrapolations from studies in Nigeria and elsewhere indicate that 5% of adult deaths could be due to hypertension [4].

A key challenge to prevention and control of CVD in Africa relates to the inability of populations in need to access trained healthcare providers and appropriate healthcare system easily and promptly, particularly in remote rural settings. As a result, detection, treatment and control rates for risk factors remain very poor at the population level, and the outcomes of care in clinical setting well below optimal [5]. The wide availability of mobile phones and the internet in Africa has provided new and inexpensive means of communication, even for those in remote rural areas. In Cameroon for instance, the majority of rural areas are covered by a GSM mobile phone network. As of January 2012, the three mobile operators in the country had over 5 million subscribers, representing a significant proportion of the total workforce. A mobile phone is often shared by the majority of households. The cost of a GSM phone call was $0.2/ minute, that of a Short Message Service (SMS) $0.1, while an hour of Internet connection cost less than $2. This may greatly reduce the cost of a telemedicine nowadays, as compared with years back when satellites communication had to be used. In addition it can simplify administrative procedures for service users. In such a context, it becomes possible for a specialized health facility in an urban area, to oversee health centers in rural areas, run by general practitioners or nurses for the management of CVD and risk factors.

Telemedicine refers to the use of telecommunication and information technologies to remotely provide clinical health care. Telemedicine is often used interchangeably with telehealth which is rather broad and extends to non-clinical services such as medical educal education, administration and research. Telehealth in general is an appealing solution to help filling the many gaps in the prevention, detection, treatment, control and monitoring of chronic diseases in resource-limited countries of Africa. However, evidence of the implementation of telehealth for chronic diseases in Africa remain very anecdotic. Therefore, the TELEMED-CAM study was designed to test whether an intervention package comprising telemedicine significantly improves control of hypertension and adherence among patients in a rural setting of in Cameroon, as compare with usual care.

## Methods

### Study settings

This was a prospective interventional study of 12 months duration from January to December 2008, in the capital city of Cameroon (Yaounde) and rural health districts within the vicinities of Yaounde. Cameroon is a central African country of about 20 million inhabitants of whom 60% live in rural areas. The population is young, with only 28% of subjects aged over 30 years. The gross national income per capita is $2060 and life expectancy at birth is 50 years. The total expenditure on health per capita is of 83 according to the World Health Statistics 2008. The prevalence of hypertension in Cameroonian adults is estimated at around 20% [6, 7].

Extrapolations from sparse reported clinical observations suggest high rates of cerebro-vascular accident (CVA), cardiac failure and chronic kidney diseases [8–11]. In rural Cameroun, the capacity of health facilities to adequately manage chronic diseases is hampered by the lack of medical doctors as well as other factors such as care costs, remoteness of the health centers and tendency of people to turn in the first place towards traditional medicine. The study protocol was approved by the Cameroon National Ethics Committee and authorized by the National Ministry of Health. Informed consent was obtained from all study participants before their enrolment.

### Participating centers

The study sites included a specialized telemedicine center and remote patient care centers. The telemedicine center was based at the Yaounde General Hospital. This is one of the two largest referral hospitals in Cameroon, with adequate technical facilities, 5 and 3 physicians specialized in the management of cardiovascular and renal diseases respectively. The study targeted the health centers within a radius of 50 to 250 km around Yaounde. Thirty eligible health centers were identified based on the following criteria: 1) Accessible by road; 2) Capability for diagnosing at least 4 new cases of hypertension per week; 3) A general practionner (GP) assuming the medical consultations; 4) A pharmacy with essential drugs, including blood pressure lowering medications; 5) Laboratory facilities for basic biological assays.

### Allocation of remote centers

Health centers were subsequently divided into two groups: an intervention group and a control group. Of the 30 eligible centers identified, six already had a pilot unit for the clinical management of hypertension and diabetes mellitus, established through a project by the Ministry of Health. These centers were therefore allocated to the control group. Of the 24 remaining centers, 10 were randomly selected and designated as intervention centers.

### Participants and power calculation

The study was powered to detect a 10 mmHg difference in systolic blood pressure (SBP), assuming a standard deviation of 18 mm Hg, a 2-sided p-value of 0.01 and a power of 90%, resulting in a minimum of 96 participants per study arm. No account was made for possible clustering effects within participating centers. Participants were recruited consecutively and concurrently at participating centers. All adult subjects (or individuals aged 15 years and above) with hypertension not at target level (SBP (or DBP) > = 140 (90) mmHg or > = 130 (80) mmHg (for those with diabetes or nephropathy) were invited to take part of the study, with no restriction regarding gender, religion ethnicity and race. In addition, they had to totalize at least 12 months of continuous residence in the study areas prior to the study. Pregnant women and patients presenting with severe target organ damage or any other severe disability were also excluded.

### Measurements and definitions

Blood pressure was measured following the procedures developed for the International Collaborative Study on Hypertension in Blacks. Briefly, after emptying their bladder, participants were seated for at least 10 minutes and during this period were queried about consumption of food, alcohol, coffee, or cigarettes within the previous 30 minutes. The arm circumference at the midpoint was measured, and an appropriate cuff (13×23 cm or 16×30 cm) was selected. The radial pulse was located, the pulse obliteration pressure was estimated, and 30 mmHg was added to this value to serve as the maximum inflation point. Systolic (SBP) and diastolic blood (DBP) pressures were measured with a standard mercury manometer three times as the first-and fifth-phase Korotkoff's sounds. Examiners were trained to deflate the mercury at a rate of 2 mm per second and record values to the closest even integer. Thirty second pulse was counted before each of the three readings. The mean of two last readings was used as the value for analysis [6]. Newly diagnosed hypertension was defined as SBP (and or DBP) > = 140 (90) mmHg in a participant not previously diagnosed with hypertension; and hypertension not at target levels defined as SBP (or DBP) > = 140 (90) mmHg or > = 130 (80) mmHg (for those with diabetes or nephropathy) in participants previously diagnosed and treated for hypertension.

Weight was recorded to the nearest 0.1 kg, by using an automated scale, after patients removed shoes and any heavy clothing. Height (in meters to the nearest 0.5cm) was measured using a rigid stadiometer displayed against a vertical wall. Waist circumference was measured midway between the iliac crest and the lower rib margin and the hip circumference was measured at the intertrochanteric level.

### Training of investigators

Specialized physicians (5 cardiologists and 3 nephrologists) from the center of telemedicine were trained to use the computer software for remote monitoring of patients as well as treatment and telecommunication protocols. The GPs of the intervention centers and their nurses were trained on patient education, completion of study forms and patient's files, measurement of clinical and follow up parameters, treatment initiation and titration, and telecommunication protocols. GPs and nurses from the control centers were not trained on treatment initiation and titration and telecommunication protocols.

### Intervention and follow-up

The usual care was applied to the control group which involved prescription of generic and non-generic blood pressure lowering medications as per routine practice in each participation control center. In the intervention group, only generic blood pressure lowering medications were allowed to be prescribed. Six pharmacological classes were available: Diuretics (hydrochlorothiazide-HCTZ), centrally acting sympatholytics (alphamethyl-dopa, clonidine), peripherally acting sympatholytics (reserpine), beta-blockers (atenolol, propanolol), calcium channel blockers (nifedipine) and angiotensin converting enzyme inhibitors (captopril).

The targeted blood pressure was SBP(and DBP) < 140 (90) mmHg (90) in people without diabetes or < 130 (80) mmHg in people with diabetes or real disease. Non pharmacological intervention was applied to all participants in the intervention group. This included weight reduction in overweight and obese patients, dietary sodium reduction, smoking cessation, decreasing alcohol reduction and increasing physical activity. In those patients who could not achieve target BP control on life style measures alone, drug therapy was introduced and titrated as appropriate to lower BP adequately. Unless contraindicated, patients were all started on a bitherapy comprising HCT and any member of the 5 other classes. In those with diabetes or kidney disease, preference was given to ACE inhibitors. Patients were seen every two weeks until the end of the study with a total of 12 visits. The primary study outcome was any change in SBP and/or DBP levels between the final and baseline visits. Secondary outcomes included the percentage of patients achieving target BP control and adherence.

### Telecare intervention

Dedicated staff from the telemedicine center was available to the peripheral centers all working days, according to a schedule. In this study, the GSM mobile telephone communication was used, given its availability and the relatively low cost of its implementation over the Internet. Therefore, each center in the intervention group was provided with a mobile phone, according to the operator offering the best conditions of fluidity of the network in its area. Patients in remote centers allocated in the intervention group were treated following standardized protocols. At any stage, staff in those centers were also allowed to liaise with the telecare center (Yaounde General Hospital) vian mobile phone for further guidance on their decision making. Futhermore, they were requested to send patients data to the telemedicine center everyday via SMS or voice mail. Request received spontaneously or as part of the daily reporting from the intervention centers were precessed without further delay by the telemedicine center with immediate realtime feedback (phone call) to the remote center for relevant action. The overall study strategy is summarized in Table 1.

**Table 1:** Overall strategy

Step	Control group	Intervention group	Telemanagement
**0**	Health centers are selected.	Health centers are selected.	The telemedicine center is well equipped and fully functional.
**1**	Training of staff on parameters measurements, data coding and filling of the questionnaires.	Training of staff on parameters measurements, data coding and filling of the questionnaires. Training of the care provider (medical doctor or nurse) on treatment protocols and patient education.	Training of the team on computer data logging and telecommunication protocols.
**2**	Forms, medical equipment and phones are provided. Start of the study.	Forms, medical equipment and phones are provided. Start of the study.	Staff schedule established Start of the study.
**3**	Recording of every newly diagnosed hypertension case	Recording of every newly diagnosed hypertension case	The center is available for direct telephone calls from the care providers of the intervention group only.
**4**	Patient receives usual care. No interaction with the telemedicine center.	Patient receives a standardized care + education. In case of a particular need, a phone call can be sent to the telemedicine centre in Yaounde.	Cases from the intervention sites are reviewed immediately at the telemedicine center and a feedback is sent to the care provider for approval or modification of the treatment within some few minutes.
**5**	After a patient consultation, his data form is logged by the nurse and kept.	After a patient consultation, his data form is logged by the nurse and kept.	Data are logged in the computer and plotted, to allow visual computerized follow-up of cases and a monitoring of the progression of the study.
**6**	Every week, data are requested from Yaounde and sent by ordinary postal mail or by car.	Every day, data are requested from Yaounde and sent by SMS or voice mail.	Steps 3, 4, 5 are repeated until the end of the study.
**7**	Steps 4, 5, 6 are repeated until the end of the study.	Feedback is received from Yaounde immediately, and treatment adjusted accordingly (up titration, adding a new agent). This interaction is done in real-time. Steps 4, 5, 6, 7 are repeated until the end of the study.	
**8**	End of study Hard copies are collected.	End of study Hard copies are collected.	Hard copies are collected. Cross check of data is done. Start of data analysis.

### Statistical analysis

All statistical analyses were performed with the use of SPSS software, version 13.0 (SPSS Inc., Chicago, IL 60606-6412). The intention-to-treat analysis included all patients who had at least one evaluation available after the first visit. In case of missing data, the last observation was carried forward. The difference between the mean number of medical visits, the mean systolic and diastolic BP in both groups areas were analyzed using a two independent samples Student's t-test. The difference of the hypertension control rate between the two groups was analyzed using a chi square test.

## Results

### Baseline characteristics and follow-up

A total of 268 participants (165 in the intervention group) were recruited in the study. These participants included more female, similarly across groups (p = 0.08). Participants in the intervention group included fewer diabetics (9.1% vs. 43.6%, p = 0.02) and were more likely to have higher SBP (169 vs. 161 mm Hg, p = 0.01) and higher DBP (100 vs. 95 mm Hg, p = 0.01), with however no effect on the distribution of participants by grade of hypertension across groups (p = 0.29, Table 2). Other baseline characteristics of participants are summarized in Table 2, largely showing similar profile across groups at baseline.

**Table 2:** Baseline characteristics of the study population

Variables	Usual care group	Intervention group	p
**N**	**103**	**165**	
**Sex**			0.08
Male, n (%)	47 (45.5)	60 (36.2)	
Female, n (%)	56 (54.5)	105 (63.8)	
Mean age, years (SD)	57.6 (12.1)	59.9 (10.4)	0.06
Diabetes mellitus, n (%)	45 (43.6)	15 (9.1)	0.01
Mean height, cm (SD)	161.5 (18.9)	165.5 (7.8)	0.02
Mean weight, Kg (SD)	75.4 (15.2)	75.4 (17.0)	0.99
Mean waist circumference, cm (SD)	93.0 (19.4)	92.1 (16.0)	0.66
Mean body mass index, kg/m^2^ (SD)	29.4 (12.6)	27.3 (6.4)	0.09
Mean systolic blood pressure, mmHg (SD)	160.8 (23.7)	169.2 (27.9)	0.01
Mean diastolic blood pressure, mmHg (SD)	95.2 (14.8)	100.4 (18.3)	0.01
Mean heart rate, beats/minute (SD)	73.8 (14.5)	76.6 (13.8)	0.12
**Hypertension**			0.29
Grade I, n (%)	8 (7.8)	18 (10.9)	
Grade II, n (%)	26 (25.0)	52 (31.5)	
Grade III, n (%)	69 (67.0)	95 (57.6)	
**Mean number of visits attended by hypertension grade (SD)**			
Grade I	3.5 (2.1)	5.2 (1.8)	0.04
Grade II	4.7 (1.7)	5.5 (1.2)	0.02
Grade III	4.5 (1.9)	5.6 (1.4)	0.01
**Blood pressure lowering medication at the last visit**			
Calcium channel blockers, n (%)	18 (17.4	25 (15.2)	0.61
Centrally acting sympatholytics, n (%)	1 (0.9)	3 (1.7)	>0.99
Beta blockers, n (%)	3 (2.7)	6 (3.4)	>0.99
Angiotensin converting enzyme inhibitors, n (%)	10 (10.1)	33 (20.3)	0.03
Diuretics (hydrochlorothiazide), n (%)	71 (68.8)	98 (59.3)	0.12

SD: standard deviation

The total duration of intervention was 24 weeks (12 post baseline visits). Regardless of the severity of hypertension, adherence to medical visits was always significantly higher among participants in the intervention group, compared to those in the usual care group (all p < = 0.04, Table 2). The pattern of BP lowering medication prescription at the last study visit is showed in Table 2. This pattern was largely similar across study groups. The most prescribed drugs in both arms were diuretics, calcium channel blockers and angiotensin converting enzyme inhibitors. Beta blockers and centrally acting sympatholytics were rarely prescribed. In all, 1187 calls were received by telemedicine center from remote clinics in the intervention groups during the study period; 791 unique provider’ call were made. The estimated average daily number of call per provider was 6, for a duration of about 5 minutes each.

### Outcomes

**Figure 1:**
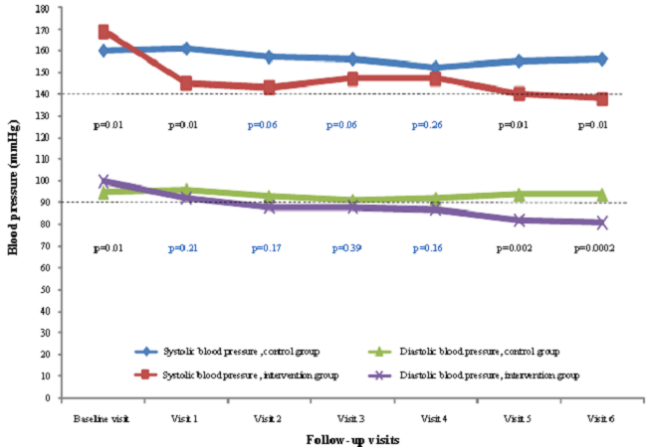
Changes in blood pressure during medical visits in both groups

**Figure 2:**
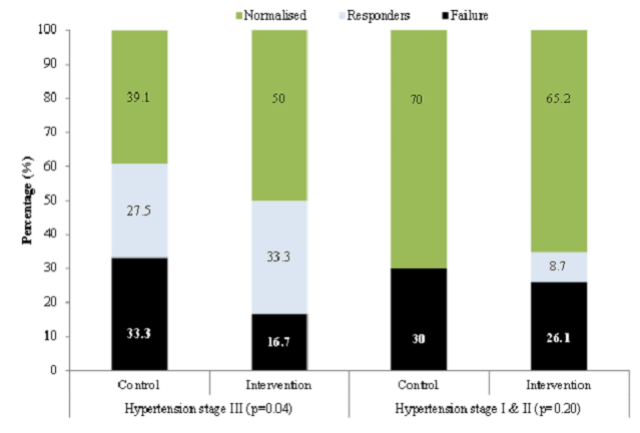
Blood pressure control at the end of the study period, depending on the severity of hypertension

Changes in blood pressure during follow-up are depicted in Figure 1. At baseline average SBP was significantly higher among participants in the intervention group as compared with those in the control group while DBP was significantly lower (both p = 0.01). During follow up and right from the second visit, SBP significantly decrease in the intervention group while remaining constant in the control group (Figure 1). The pattern was somehow different for DBP with levels remaining mostly similar across intervention arm, with a through separation observed only during the last two visits. By then, DBP was lower in the control group than in the intervention group (Figure 1). At the final visit, there was differential control rate of hypertension across treatment allocation groups according to the severity of hypertension at baseline. For instance, among participants with stage 3 hypertension at baseline, 50% in the intervention group and 39.1% in the usual car group were at target BP levels at the final visit. A further 33.3% of participants in the intervention group (27.5% in the usual care group) not at target, had improved BP levels while only 16.7% in the control groups against 33.3% in the usual care group had no improvement in BP level between baseline and final visits (Figure 2, p = 0.04). By comparison, among participants with hypertension stage I-II at baseline, nearly similar proportions in the intervention (65.2%) and in the control groups (70%) were at target BP levels at the final visit (Figure 2, p = 0.20).

## Discussion

Telemedicine has been advocated as an innovative mean for improving both access to- and quality of health care in developing countries [12]. In this pilot TELEMED-CAM project, we have demonstrated the significant beneficial effect of an intervention package comprising telecare support on average blood pressure levels and control rates for hypertension among adults Cameroonian receiving ongoing chronic care in rural health facilities. Interestingly, these effects were apparent early in the course of the intervention, and sustained throughout the whole duration of the study.

The application of telemedicine to chronic diseases management has been around for over twenty years now, and has been used both in the process of care and outcome of care [13]. With regard to hypertension care, the efficacy of telemedicine in the management has been tested in clinical trials. The wide range of telemedicine applications trialed makes comparison across studies very challenging. However, the balance of the available evidence tends to support the effectiveness of telemedicine in reducing blood pressure levels and improving hypertension control [13]. A recent systematic review of twenty randomized controlled trials found that home BP tele-monitoring (HBPT) for instance was associated with a significant 4.7 mmHg and 2.4 mmHg reduction in SBP and DBP respectively [14]. Furthermore, more patients in the intervention group achieved optimal office blood pressure control [14], which is in line with our finding of a significantly higher proportion of patients reaching the desired target blood pressure levels at the end of follow-up in our study. Previous application of telemedicine for chronic disease in the study setting has been in the area of tele-diagnostic of chronic complications [15]. Apparently, the application of telemedicine to chronic non-communicable disease in Africa in general is still at an embryonic stage.

Telemedicine interactions in studies have been essentially of two types: 1) the real-time approach (e.g. videoconferencing, telephone, etc.) and the asynchronous approach (store-and-forward transmission of data for instance). Our study used a complex approach integrating both real-time and asynchronous interactions. In such an approach imposed in major ways by the realities of the study setting, it would be very difficult to assess the contribution of each component of the interactions to the outcome of the intervention. Furthermore, applications of telemedicine to chronic disease management have focused on interventions which relate the patients directly to the telecare center [13]. Our study focused on providing tele-support to healthcare provider in charge of the management of patients with hypertension in rural settings, with otherwise, no access to specialized care. Previous efforts to improve access to hypertension and chronic disease management in rural Cameroon have involved task-delegation through setting-up nurse-led clinics [16, 17]. In this past experience however, nurses’ efforts were monitored and strengthened via regular site visits by the supervision team, which may substantially increase the cost of the intervention [18]. Findings from the current study fits directly into the context-specific knowledge base for managing hypertension, by suggesting that remote supervision of nurses- or GP-led hypertension clinics in rural settings, is possible via telemedicine, while maintaining the effectiveness of the intervention, as found elsewhere [19, 20]. Experience from developed countries suggests that even the training component of the intervention can in major ways be effectively delivered remotely [21]. However, the overall cost of implementing such a system has to be assessed [14].

Cardiovascular disease is a major public health problem in Cameroon and over the last ten years, there have been a number of initiatives aimed at scaling up the fight against chronic diseases in general, and hypertension and diabetes mellitus in particular in the country [22], including: 1) the adoption of a National Strategy for hypertension and diabetes [22]; and 2) the development of training and task-shifting programmes to improve detection and management at primary care level [16, 17]. In spite of these efforts, recent studies showed low awareness, treatment and control rates in the context of escalating prevalence of hypertension [5]. These findings tend to suggest that the good will contained in the National Strategy has not yet been fully translated into actions with sizable positive effects on the health of the population. This low awareness and control rate of hypertension has been reported as a global phenomenon [23, 24]. However, possible constraints to the translation of the national strategy to actions in Cameroon would likely include the rising incidence of the disease as reflected by the epidemiological transition in this part of the world, but also the shortage of health manpower with a very low and inadequate patient/doctor ratio in the country. Therefore, innovative approaches are needed to rapidly expand the health workforce.

With an improvement of blood pressure control in the intervention group as compared to the control group, our study showed that an intervention package comprising telemedicine is a potential alternative strategy for improving access to adequate care for chronic disease in the context of limited human resources. Our findings also suggest that integrating telemedicine system could contribute to improving universal access to health and to strengthening the weakened health care systems. As proven elsewhere [25], another impact of this study is for policy and decision makers and politicians, given the potential contribution of this type of intervention to health care improvement as highlighted in the UN Millennium Development Goals (MDGs) framework and by many other organizations [26]. The last but not the least advantage of telemedicine in low-resource settings is to provide decision makers with timely, reliable and standardized data for resource allocation, effective drug supply and management.

Telemedicine relies on information communication technologies (ICT) which are not readymade fit. ICT always needs to be contextualized and above all needs a commitment to be used, thus capacity building and the process of creating an informed society are crucial for its implementation. In Cameroon and many sub-Saharan African countries low bandwidth, slow connections and high service charges are some of the current technological challenges. Moreover, the high cost of telecommunication services, competing with other basic priorities, such as food, clothing, school fees, health needs in the absence of an insurance policy, makes internet and mobile phones unaffordable for a large segment of the population. However the recent trend of increasing penetration rates of mobile technology in the developing world, especially in Africa, can be considered as an opportunity to implement applications at the grass roots level by empowering community-based health care workers, using simple, relevant, and combined technology with local content and language interface [27].

Limitations of this study include the non-strictly random allocation of centers/participants, the unblinded assessment of end-points and the implementation by a few practitioners in relatively very few places, so that the findings cannot be generalized. The geographical contiguity of participating health districts made rigorous application of the clustering design very challenging as the risk of contamination would be very high; and we did not account for the clustering effect in the sample size estimation and data analysis. Furthermore, we did not collect key information to assess the impact of tele-intervention, such as the number of treatment plans changed as the results of the interaction between remote clinics and our telemedicine. Lastly, telehealth was just a component of an intervention package in this study, since other procedures were applied to centers and participants in the intervention groups, and which could possibly have a beneficial effect on blood pressure control and contribute to so of the positive effect observed in our study. These additional procedures include the selective standardization of treatment protocol in the intervention group, selective provision of education materials or selective training of healthcare providers in the intervention group on the use of treatment protocols. Therefore, the observed reduction in blood pressure in the intervention group in the current study likely reflects the combined effects of all those procedures and not the sole effect of telehealth alone. In spite of these limitations, this first report on the application of telemedicine for hypertension management in Cameroon and potentially in most of the sub-Saharan African region, adds to our knowledge by demonstrating findings suggesting that telehealth may have several applications for chronic disease care in the country/region.

## Conclusion

Our study demonstrated the significant beneficial effect of an intervention package comprising telecare support on average blood pressure levels and control rates for hypertension among adults Cameroonian receiving ongoing chronic care in rural health facilities. The application of ICT and telemedicine therefore appear as an alternative and feasible strategy to improve access to care and the outcome of care for hypertension in resources-limited settings. Further research is needed on the context-sensitive approaches to scale up beyond proof of concept, the evaluation of impact and cost of implementation.

## References

[1] World Health Organisation (2002). The World health report 2002: Reducing risk, promoting healthy life.

[2] Reddy KS, Yusuf S (1998). Emerging epidemic of cardiovascular disease in developing countries. Circulation..

[3] Kadiri S (2005). Tackling cardiovascular disease in Africa. BMJ..

[4] Cooper RS, Rotimi CN, Kaufman JS, Muna WF, Mensah GA (1998). Hypertension treatment and control in sub-Saharan Africa: the epidemiological basis for policy. BMJ..

[5] Dzudie A, Kengne AP, Muna WF, Ba H, Menanga A, Kouam Kouam C (2012). Prevalence, awareness, treatment and control of hypertension in a self-selected sub-Saharan African urban population: a cross-sectional study. BMJ Open..

[6] Cooper R, Rotimi C, Ataman S, McGee D, Osotimehin B, Kadiri S (1997). The prevalence of hypertension in seven populations of west African origin. Am J Public Health..

[7] Mbanya JC, Minkoulou EM, Salah JN, Balkau B (1998). The prevalence of hypertension in rural and urban Cameroon. Int J Epidemiol..

[8] Muna WF (1993). Cardiovascular disorders in Africa. World Health Stat Q..

[9] Kengne AP, Anderson CS (2006). The neglected burden of stroke in Sub-Saharan Africa. Int J Stroke..

[10] Kingue S, Dzudie A, Menanga A, Akono M, Ouankou M, Muna W (2005). A new look at adult chronic heart failure in Africa in the age of the Doppler echocardiography: experience of the medicine department at Yaounde General Hospital. Ann Cardiol Angeiol (Paris).

[11] Djoumessi S, Nouedoui C, Youmbissi TJ, Essengue Behl GF (2000). Biological markers of renal dysfunction in essential arterial hypertension in African subjects]. Ann Biol Clin (Paris).

[12] Kaplan WA (2006). Can the ubiquitous power of mobile phones be used to improve health outcomes in developing countries?. Globalization and health..

[13] Wootton R (2012). Twenty years of telemedicine in chronic disease management--an evidence synthesis. J Telemed Telecare..

[14] Omboni S, Gazzola T, Carabelli G, Parati G (2013). Clinical usefulness and cost effectiveness of home blood pressure telemonitoring: meta-analysis of randomized controlled studies. J Hypertens..

[15] Jivraj I, Ng M, Rudnisky CJ, Dimla B, Tambe E, Nathoo N (2011). Prevalence and severity of diabetic retinopathy in Northwest Cameroon as identified by teleophthalmology. Telemed J E Health..

[16] Kengne AP, Sobngwi E, Fezeu L, Awah KP, Dongmo S, Mbanya JC (2009). Setting-up nurse-led pilot clinics for the management of non-communicable diseases at primary health care level in resource-limited settings of Africa. PAMJ..

[17] Lekoubou A, Awah P, Fezeu L, Sobngwi E, Kengne AP (2010). Hypertension, diabetes mellitus and task shifting in their management in sub-Saharan Africa. Int J Environ Res Public Health..

[18] Kengne AP, Awah PK, Fezeu LL, Sobngwi E, Mbanya JC (2009). Primary health care for hypertension by nurses in rural and urban sub-Saharan Africa. J Clin Hypertens (Greenwich).

[19] Bove AA, Santamore WP, Homko C, Kashem A, Cross R, McConnell TR (2011). Reducing cardiovascular disease risk in medically underserved urban and rural communities. Am Heart J..

[20] Scalvini S, Rivadossi F, Comini L, Muiesan ML, Glisenti F (2011). Telemedicine: the role of specialist second opinion for GPs in the care of hypertensive patients. Blood Press..

[21] Masi C, Hamlish T, Davis A, Bordenave K, Brown S, Perea B (2012). Using an established telehealth model to train urban primary care providers on hypertension management. J Clin Hypertens (Greenwich).

[22] Njamnshi AK, Bella Hiag A, Mbanya JC (2006). From research to policy: the development of a national diabetes programme in Cameroon. Diabetes Voice..

[23] Hendriks ME, Wit FW, Roos MT, Brewster LM, Akande TM, de Beer IH (2012). Hypertension in sub-Saharan Africa: cross-sectional surveys in four rural and urban communities. PLoS One..

[24] de Ramirez SS, Enquobahrie DA, Nyadzi G, Mjungu D, Magombo F, Ramirez M (2010). Prevalence and correlates of hypertension: a cross-sectional study among rural populations in sub-Saharan Africa. J Hum Hypertens..

[25] Bagayoko CO, Anne A, Fieschi M, Geissbuhler A (2011). Can ICTs Contribute to the Efficiency and Provide Equitable Access to the Health Care System in Sub-Saharan Africa?. The Mali Experience. Yearb Med Inform..

[26] Dyer O (2005). UN predicts that millennium development goals will be missed by a wide margin in Africa. BMJ..

[27] Geissbuhler A, Ly O, Lovis C, L'Haire JF (2003). Telemedicine in Western Africa: lessons learned from a pilot project in Mali, perspectives and recommendations. AMIA Annu Symp Proc..

